# KCl-Dependent Release of Mitochondrial Membrane-Bound Arginase Appears to Be a Novel Variant of Arginase-II

**DOI:** 10.1155/2016/3675283

**Published:** 2016-05-16

**Authors:** Mishra Suman, Mishra Rajnikant

**Affiliations:** Biochemistry and Molecular Biology Laboratory, Department of Zoology, Banaras Hindu University, Varanasi 221005, India

## Abstract

Arginase regulates arginine metabolism, ornithine-urea cycle, and immunological surveillance. Arginase-I is predominant in cytosol, and arginase-II is localised in the mitochondria. A mitochondrial membrane-bound arginase has also been proposed to be adsorbed with outer membrane of mitochondria which gets released by 150 mM potassium chloride (KCl). It is presumed that inclusion of 150 mM KCl in the homogenization medium would not only facilitate release of arginase bound with outer membrane of mitochondria but also affect functional anatomy of mitochondria, mitochondrial enzymes, and proteins. Therefore, it has been intended to characterize KCl-dependent release of mitochondrial membrane-bound arginase from liver of mice. Results provide advancement in the area of arginase biology and suggest that fraction of mitochondrial membrane-bound arginase contains mitochondrial arginase-II and a variant of arginase-II.

## 1. Introduction

Arginase [E.C 3.5.3.1] catalyzes synthesis of ornithine and urea from arginine [1]. It regulates level of L-arginine [2] and production of nitrous oxide (NO) [3]. It has also been important for immunological surveillance [4, 5]. It gets upregulated in vascular abnormalities [6, 7], diabetes [8], hepatocellular carcinoma [9], cardiovascular diseases [10], and neuroinflammation [11]. Other roles of arginase have also been reviewed [12], showing impact of arginase on biological significance of arginine metabolism associated pathological conditions. Two major isoforms of arginase, cytosolic arginase-I and mitochondrial arginase-II, have been described in different organisms [13–16] as having distinct physicochemical, molecular, and immunological properties. Several other isoforms have also been reported on the basis of difference in net-charge, immunological properties, and subcellular localization. Three isoforms of arginase have been observed in rat liver on the basis of elution pattern of CM-cellulose chromatography [17] and electrophoresis pattern [18]. Four isoforms of arginase in frog and lizard on the basis of DEAE-cellulose chromatography [19] and five isoforms of arginase in rat on the basis of immunological properties [20] have also been proposed. One of the forms of arginase has also been proposed to bind with external side of inner mitochondrial membrane of chicken kidney [21]. However, cytosolic arginase-I has also been thought to be associated at the surface of subcellular organelles [22]. The activity of arginase has also been observed [23–25] in the fractions extracted with 150 mM potassium chloride (KCl) or butanol [26]. The use of KCl in homogenizing medium for isolation of mitochondria has resulted in increased arginase activity in cytosolic fraction and decreased arginase activity in mitochondrial fraction [25]. However, the high salt concentration [27, 28] may affect mitochondrial membrane. It is presumed that 150 mM KCl may not only facilitate release of arginase bound with membrane of mitochondria but also affect integrity of mitochondria and other cell organelles. It is likely that arginase-II gets released from mitochondria due to alterations in permeability or damage of mitochondrial membrane under nonoptimal KCl concentration. The posttranslational modification of arginase-II may also alter targeting or translocation to the mitochondria that may remain adhered either to outer or to inner membrane (Figure 8) of mitochondria. The activity of mitochondrial membrane-bound arginase has been indicated but biochemical and molecular characterization of the mitochondrial membrane-bound arginase is limiting. Therefore, it has been intended to evaluate KCl-dependent activity of mitochondrial membrane-bound arginase from mitochondria isolated from mice liver. The results suggest that KCl-dependent release of mitochondrial membrane-bound arginase appears to be a variant of arginase-II.

## 2. Materials and Methods

### 2.1. Animals and Materials

The adult (12 ± 2 weeks) mice (*Mus musculus*) of AKR strain were maintained with standard mice feed and drinking water at 25 ± 2°C in animal house facility of the department as per guidelines of the Institutional Animal Ethical Committee. Animals were sacrificed to obtain liver. All analytical grade chemicals were used and purchased from local suppliers. Anti-arginase-I (sc-20150), anti-arginase-II (sc-20151), COX II (sc-23983), and anti-GAPDH (sc-25778) were purchased from Santa-Cruz Biotechnology Inc. (USA) and anti-cytochrome C (C5723) was purchased from Sigma-Aldrich (USA). They are well characterized and widely used antibodies in the literature.

### 2.2. Effect of KCl on Mitochondrial Membrane Associated Arginase

**Figure 1:**
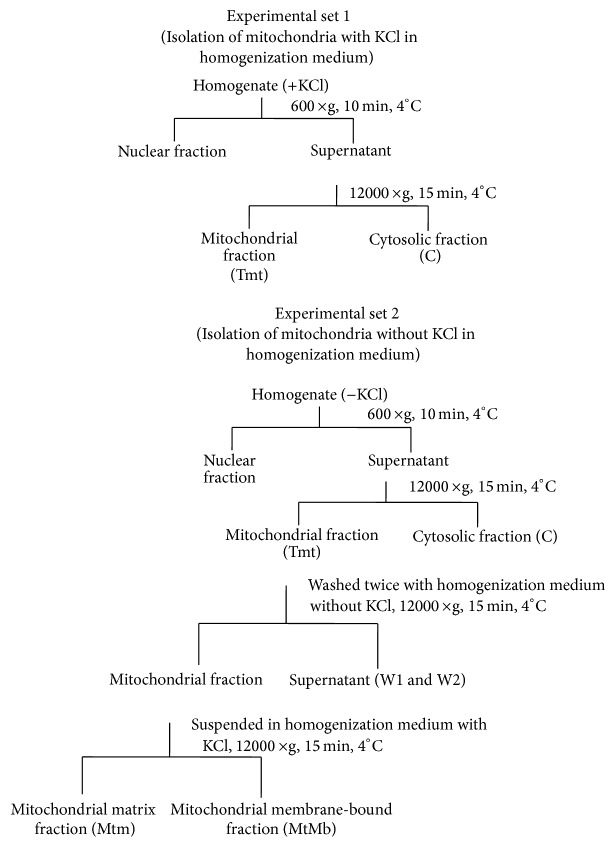
Flow chart of the procedure of isolation of cytosolic fraction, mitochondrial fraction, and mitochondrial outer membrane-bound fraction. The experimental set 1 mitochondria were isolated with KCl in homogenizing medium and experiment set 2 in which mitochondria were isolated first and then washed with different concentration of KCl in homogenizing buffer.

The experimental plan of isolation of cytosol, mitochondria, and mitochondrial membrane-bound arginase was summarized in Figure 1. In brief, 20% homogenate of liver tissue was prepared in homogenizing buffer containing 30 mM Tris HCl (pH 7.2), 1 mM EDTA, 250 mM sucrose, 50 mM mannitol, and protease inhibitor cocktail (Sigma-Aldrich) using a Potter-Elvehjem type glass homogenizer with a motor-driven teflon pestle. Homogenate was centrifuged at 600 ×g for 10 min and pellet was discarded. The supernatant was further centrifuged at 12,000 ×g for 15 min for isolation of mitochondria. The supernatant was considered as cytosolic fraction (C) and pellet was washed twice with homogenizing buffer (without KCl) and centrifuged at 12,000 ×g for 15 min. Both supernatants (W1 and W2) were analysed to confirm the release of mitochondrial matrix arginase or contamination of cytosolic arginase in mitochondrial pellet. The pellet obtained after second wash (W2) was considered as total mitochondrial pellet (Tmt). For the isolation of mitochondrial membrane-bound fraction, the washed mitochondrial pellet (Tmt) was suspended with KCl-containing homogenizing buffer (100 mM KCl) and centrifuged at 12,000 ×g for 15 min. The supernatant was considered as the mitochondrial membrane-bound fraction (MtMb) and the pellet as the washed mitochondrial matrix fraction (Mtm).

The first set of experiment was like earlier reports to evaluate effects of KCl when used in homogenization medium before isolation of mitochondria. In first set, different concentrations of KCl (0, 25, 50, 100, 150, and 200 mM) were used in homogenizing medium, and mitochondrial and cytosolic fractions (C) were isolated. The second set of experiment was planned to confirm that the activity of arginase was due to KCl treatment on the mitochondrial membrane, not contributed by membrane of any other subcellular organelle. In second set, mitochondria were isolated first without KCl in homogenizing medium. Washed mitochondrial pellet (total mitochondria, Tmt) was suspended in different concentrations of KCl (0, 25, 50, 100, 150, and 200 mM) in homogenizing medium. Then, membrane-bound (MtMb) and mitochondrial fractions (Mtm) were separated. These fractions were used for arginase assay and western blot analysis. The 100 mM KCl was used in validation of subcellular fractionation.

The extraction of cytosolic, mitochondrial, and membrane-bound fraction was evaluated by mitochondrial marker enzyme assay. The activity of succinate dehydrogenase (SDH) was measured by the spectrophotometric method of Ells [29] in 1 mL reaction mixture containing 67.5 mM potassium phosphate buffer (pH 8.5), 16.6 mM succinate, 2 mM nicotinamide adenine dinucleotide (NAD^+^), 2 mM phenazine methosulfate (PMS), and 2,6-dichlorophenolindophenol (DCPIP). The continuous assay started after addition of enzyme source and absorbance was recorded at 600 nm up to 3 min. Difference in absorbance was used for the calculation of activity. One unit of SDH activity was expressed as the amount that utilized 1 *μ*mole of NAD^+^ per min at 30°C. Protein was estimated by the method of Bradford [30] taking bovine serum albumin as standard.

Arginase assay was performed according to the method of Brown Jr. and Cohen [31]. The reaction mixture consisting of 25 mM sodium glycinate buffer (pH 9.5), 2.5 mM MnCl_2_, 25 mM L-arginine, and suitably diluted enzyme extract, with total volume of 2 mL, was incubated for 15 minutes at 30°C. The reaction was terminated by adding 10% perchloric acid, and protein was removed by centrifugation. Urea was determined in the supernatant. One unit of arginase activity was defined as the amount of enzyme, which produced one *μ*mole of urea per hour at 30°C.

### 2.3. Analysis of Expression Pattern of Arginase-I and Arginase-II by Western Blot and RT-PCR

Proteins of cytosolic, mitochondrial, and mitochondrial membrane-bound fractions were resolved on 15% denaturing gel. Resolved polypeptides were transferred on polyvinylidene difluoride (PVDF) membrane. Membrane was blocked with 5% nonfat milk in 1x TBS-T for one hour after the transfer of protein. After blocking, the transferred membrane was incubated with anti-arginase-I and anti-arginase-II at 1 : 500 dilutions in 2% nonfat milk in 1x TBS-T for overnight at 4°C for both. The membrane was washed four times for fifteen minutes each after primary antibody incubation. Membrane was incubated with secondary antibody (goat anti-rabbit IgG antibody, Genei) at 1 : 5000 dilutions in 2% nonfat milk in 1x TBS-T for 2 hours for all four antibodies used. Anti-cytochrome C and anti-GAPDH were used as positive controls at 1 : 1000 dilutions, respectively. For validation of subcellular fractionation, cytosolic marker GAPDH at 1 : 1000 and mitochondrial marker COX II (sc-23983) at 1 : 500 dilutions were used. Immunodetection was performed using the enhanced chemiluminescence (ECL) system. The signals of chemiluminescence were detected and analysed through LAS 500, Chemi-doc (GE Healthcare) imaging system.

Total RNA was extracted from liver using TRIzol (Invitrogen). The RNA was quantified by spectrophotometric analysis and the quality was checked on 1% agarose gel. Isolated RNA was reverse transcribed using Oligo-dT primer according to suggested protocol for first strand cDNA synthesis kit (Applied Biosystems). The RT-PCR was performed by using primers RARGIF 5′GTCCAGAAGAATGGAAGAGTCAG3′; RARGIflR 5′ CGTGGATATAGGCTACC3′; RARGIIF 5′GCACTCACTCGAGGTCCTG3′; and RARGFIIR 5′GACTCCTTCAAACTTACATG3′. The PCR products were analysed on 1% agarose gel.

### 2.4. *In Silico* Analysis of Arginase-I and Arginase-II

The putative mitochondrial targeting of arginase-I and arginase-II was also analysed* in silico*. The GenBank sequences for arginase-I (AAA98611.1) and arginase-II (AAC22548.1) were obtained from NCBI (http://www.ncbi.nlm.nih.gov/) and were studied for presumed subcellular targeting. The subcellular localization was predicted by MitoProt-II (http://ihg.gsf.de/ihg/mitoprot.html) and iPSORT (http://ipsort.hgc.jp/predict.cgi) servers.

Gene and transcript analyses of arginase-I and arginase-II were done by UCSC genome browser (https://genome.ucsc.edu/) and NCBI Ensembl (http://www.ensembl.org/) databases. EST was searched in NCBI EST database (http://www.ncbi.nlm.nih.gov/nucest/). Also, alternative splice variants were studied by ASSP (http://wangcomputing.com/assp/). The physicochemical properties and posttranslational modification were predicted by ExPAsy proteomic tool (http://www.expasy.org/proteomics) also described in Table 1.

### 2.5. Electron Microscopy of Mitochondria

The mitochondrial pellets of control were isolated without KCl and treated (isolated with 100 mM KCl, as they were found to have intermediate concentration) by the protocol of second experiment set. The mitochondrial pellets were fixed in 2.5% glutaraldehyde and 1% paraformaldehyde in phosphate buffer (pH 7.5) at 4°C overnight. The fixed mitochondrial pellets were dehydrated through grades of alcohol (30% to absolute alcohol), and the preparation of block, sectioning, and staining for TEM analysis was executed at AIIMS, New Delhi.

### 2.6. Statistical Analysis

The values are expressed as the mean ± SD. The data were analysed by one-way analysis of variance (ANOVA) followed by Tukey's test. The level of significance is taken at *P* < 0.05.

## 3. Results

### 3.1. The Cytosolic, Mitochondrial, and Mitochondrial Membrane-Bound Fractions Show Presence of Suitable Biochemical Markers

**Figure 2:**
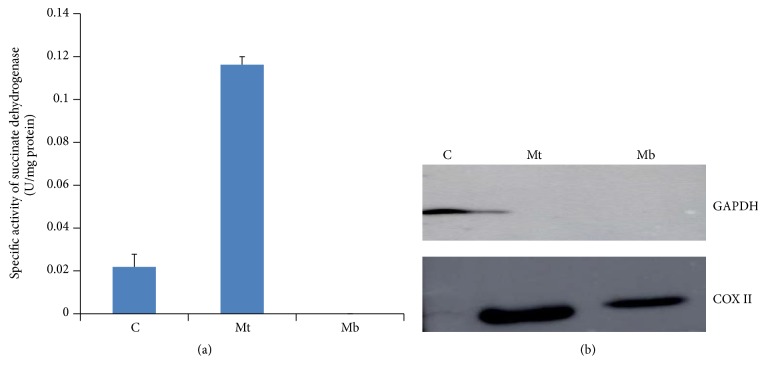
(a) Specific activity analysis of succinate dehydrogenase (SDH). (b) Western blot analysis of cytosolic GAPDH and mitochondrial COX II in C = cytosol, Mt = mitochondrial, and Mb = mitochondrial membrane-bound fraction.

The cytosolic, mitochondrial, and mitochondrial membrane-bound fractions were isolated by differential centrifugation method and validated by the spectrophotometric analysis of SDH (Figure 2(a)) and western blot analysis by GAPDH and COX II (Figure 2(b)). The spectrophotometric analysis did not detect activity in mitochondrial membrane-bound fraction but some activity was observed in cytosolic fraction, which may be due to disruption of mitochondria during handling (Figure 2(a)). The anti-GAPDH did not show any immunoreactive band in mitochondrial and mitochondrial membrane-bound fraction that could be considered as the mitochondrial fraction was free from the cytosolic contamination. The COX II detected band in mitochondrial matrix and mitochondrial membrane-bound fraction (Figure 2(b)).

### 3.2. Expression Pattern and Activities of Arginase Were Progressively Affected by KCl

**Figure 3:**
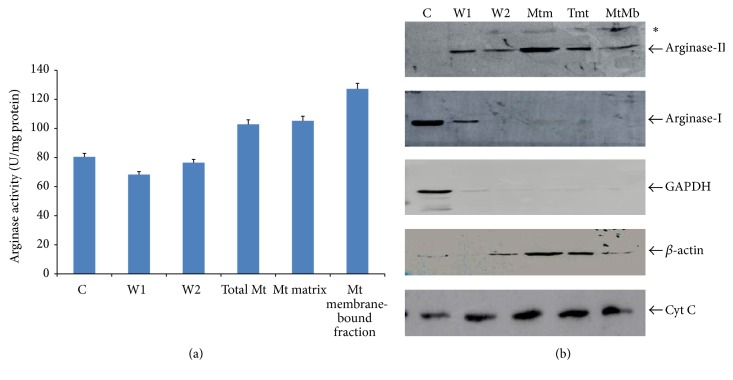
Analysis of arginase isoforms in subcellular fraction of liver of* Mus musculus*. (a) Histogram represents activity of arginase in different subcellular fractions and (b) shows western blot analysis of cytosolic fraction, total mitochondria, mitochondrial matrix, and mitochondrial outer membrane fractions probed with anti-arginase-II, anti-arginase-I, anti-GAPDH, anti-*β*-actin, and anti-cytochrome C (from top to bottom). The mitochondrial arginase was not observed in cytosolic fraction, whereas it was detected in mitochondrial outer membrane fraction (KCl-treated fraction). The anti-arginase-I did not show cross reactivity with mitochondrial fractions as well as mitochondrial membrane-bound fraction. It clearly indicates that mitochondrial membrane-bound arginase is associated with mitochondrial arginase-II. C = cytosolic fraction, W1 = first wash of total mitochondrial fraction with homogenizing medium, W2 = second wash of total mitochondrial fraction with homogenizing medium, Mtm = mitochondrial matrix fraction (after KCl wash), Tmt = total mitochondrial fraction (before KCl treatment), and MtMb = mitochondrial outer membrane fraction (KCl-washed supernatant).  ^*∗*^The variant that is immunoreactive with anti-arginase-II.

The arginase activity was detected in cytosolic fraction (C), supernatant of first mitochondrial wash (W1), supernatant of second mitochondrial wash (W2), mitochondrial matrix fraction (Mtm), total mitochondrial fraction (TMt), and mitochondrial membrane-bound fraction (MtMb). However, the maximum specific activity was observed in mitochondrial membrane-bound fraction (Figure 3(a)). Since arginase assay system could not differentiate the isoform of arginase, western blot analysis was performed in all fractions and probed with anti-arginase-I, anti-arginase-II, GAPDH, and cytochrome C. The anti-arginase-I reactive band was observed (Figure 3(b)) in cytosolic fraction but no bands were seen in mitochondrial and mitochondrial membrane-bound fraction. However, single band was detected in both washed fractions with anti-arginase-I and anti-arginase-II. The anti-arginase-II showed two immunoreactive bands in the mitochondrial membrane-bound fraction but did not detect any immunoreactive band in cytosolic fraction. One of the two bands, matched with canonical arginase-II while having an additional immune-reactive band (marked by arrow) identified by arginase-II, was observed specifically in mitochondrial fraction (Figure 3(b)).

**Figure 4 fig4:**
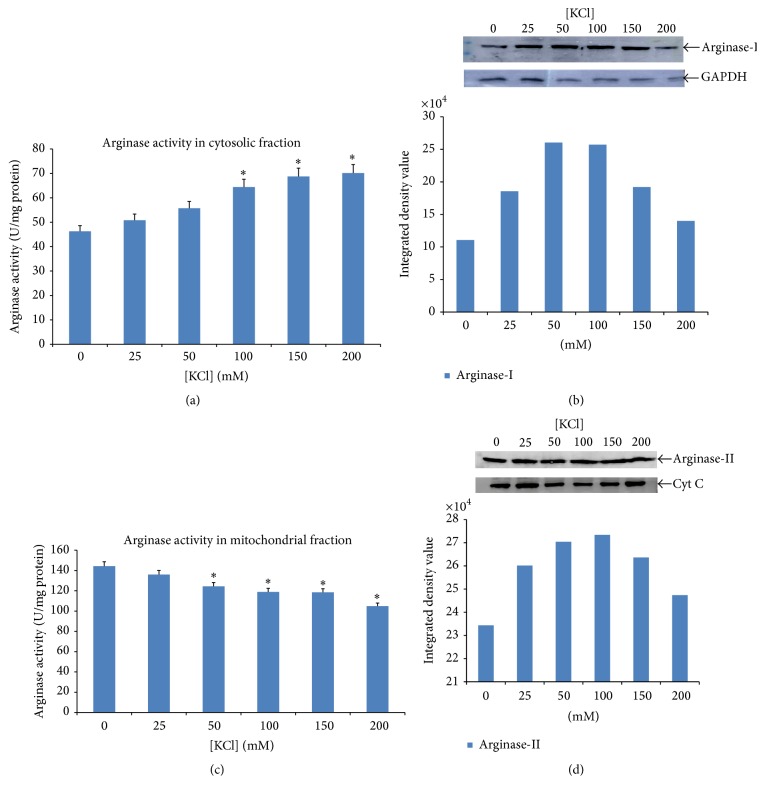
Showing impact of concentration of KCl in homogenizing medium on the activity of arginase. E1 shows the experimental set 1 described in the text. (a) The activity of arginase in cytosolic fraction. Data is mean of three experiments and histogram represents mean ± SD; ^*∗*^ *P* < 0.05. (b) The anti-arginase-I cross reacts with the single band of 40 kDa in cytosolic fraction and their activity increases with increasing KCl concentration in homogenizing medium. Glyceraldehyde 3-phosphate dehydrogenase (GAPDH) is taken as loading control. This result indicates the solubilisation of arginase-I proteins from the membrane of mitochondrial as well as other subcellular organelles. (c) The arginase activity decreases in the mitochondrial fraction with the increasing KCl concentration. But the percent decrease in the activity of the mitochondrial arginase-II is less than increase in the activity of cytosolic arginase-I; ^*∗*^ *P* < 0.05. (d) Anti-arginase-II has cross reactivity with the single band of the 40 kDa of mitochondrial fraction. The intensity of this band was decreased with increasing KCl concentration. Cytochrome C (Cyt C) was taken as loading control.

**Figure 5:**
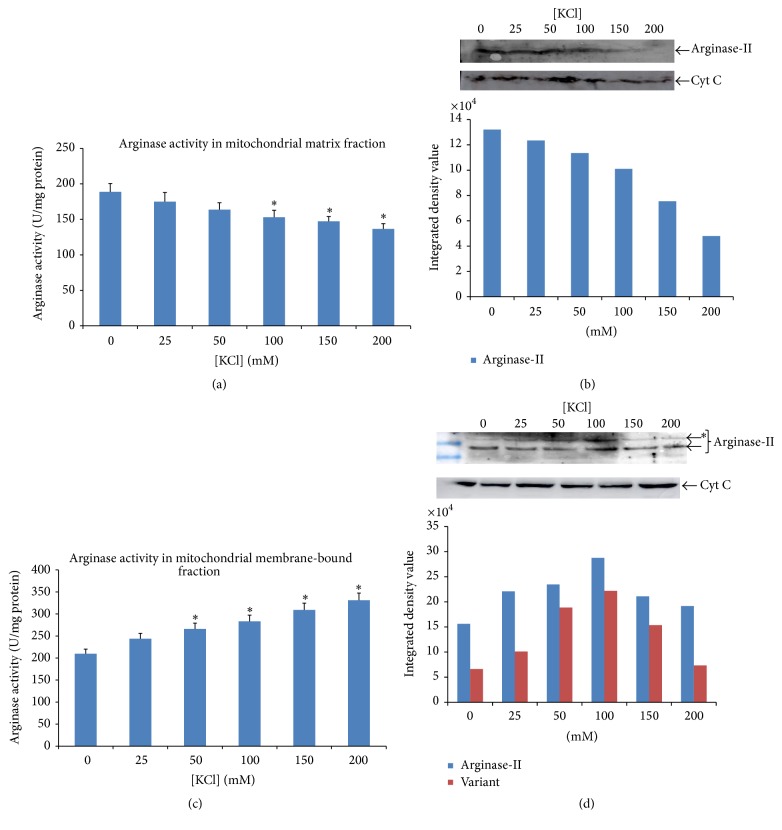
Data showing the activity of arginase under influence of increasing KCl concentration on isolated mitochondrial fraction in experiment set 2 in which mitochondria were isolated first and then washed with different concentration of KCl in homogenizing buffer. (a) Graph shows decreasing activity of arginase with the increasing concentration of KCl in the homogenizing buffer. Data is mean of three experiments and histogram represents mean ± SD; ^*∗*^ *P* < 0.05. (b) The mitochondrial matrix fraction probed against anti-arginase-II cross reacts with single band of 40 kDa. The intensity of band was decreasing with increasing concentration of KCl. Cytochrome C (Cyt C) was taken as loading control. (c) The increased activity of arginase in membrane-bound fraction was observed with increasing concentration of KCl in isolation buffer; ^*∗*^ *P* < 0.05. (d) The membrane-bound fraction probed against anti-arginase-II has shown band of 40 kDa which is expected for anti-arginase-II along with the band of 50 kDa that shows progressive increase with increasing concentration of KCl;  ^*∗*^The variant that is immunoreactive with anti-arginase-II. Cytochrome C (Cyt C) was taken as loading control.

The activity of arginase (Figure 4(a)) was observed to be progressively higher in cytosolic fraction, whereas activity of arginase in mitochondrial fraction (Figure 4(c)) was lower in experiment set 1. Similar pattern was found in western blot analysis by anti-arginase-I and anti-arginase-II in cytosol and mitochondrial fraction (Figures 4(b) and 4(d)). The activity of arginase increased in mitochondrial membrane-bound fraction (Figure 5(c)) but decreased in mitochondrial fraction (Figure 5(a)) in experiment set 2. The decrease in activity of arginase in mitochondrial fraction was similar to the increase in membrane-bound fraction. However, in the western blot analysis, an additional band with anti-arginase-II was observed which was also increasing with increasing concentration of KCl in isolation medium. Simultaneously, the intensity of expected band of anti-arginase-II was decreasing with increasing KCl concentration in isolation medium from mitochondrial fraction of experiment set 2 (Figures 5(b) and 5(d)). The percent (%) of arginase activity in mitochondrial membrane-bound fraction was >50% of the total mitochondrial arginase activity, which was also found to be increasing progressively with increasing concentration of KCl.

### 3.3. *In Silico* Analysis of Arginase and Predicted Alterations in Mitochondrial Targeting and Posttranslational Modifications of Arginase

**Figure 6 fig6:**
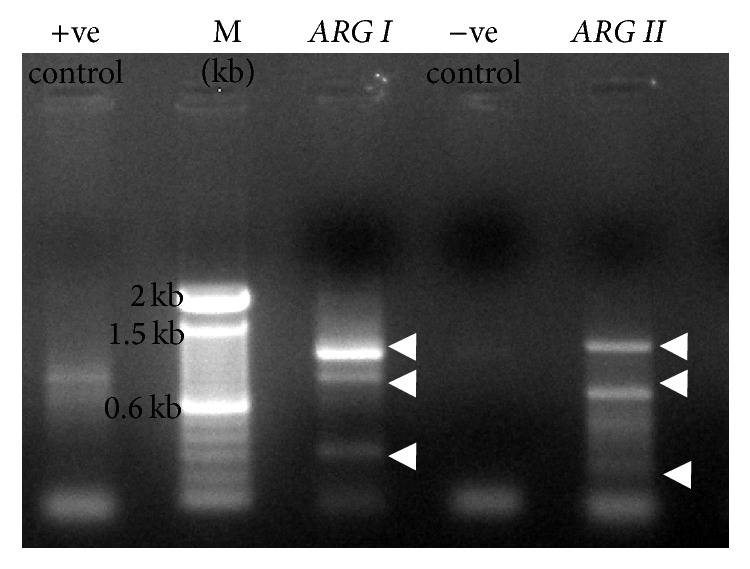
RT-PCR analysis of* ARG I* and* ARG II *transcripts. Three* ARG I* (1000 bp, 800 bp, and 300 bp) and three* ARG II* (1200 bp, 700 bp, and 300 bp) transcripts were indicated by arrow. Bp showed marker,* ARG I* = arginase-I, and* ARG II* = arginase-II. Negative control is reaction mixture without template and positive control primers provided by the manufacturer.

PCR products (1 kb, 800 bp, and 300 bp) of* ARG I* and* ARG II *(1.2 kb, 700 bp, and 300 bp) were observed in RT-PCR analysis (Figure 6). Results of western blot analysis and RT-PCR indicated variants of arginase and modulation of arginase-I and arginase-II. The* in silico* prediction of posttranslational modification of arginase suggests the probability of phosphorylation at serine and threonine residues (http://www.expasy.org/proteomics). Not only phosphorylation but also glycosylation sites were found in arginase-II protein sequence. These modifications may alter the mobility of arginase and association with mitochondrial membrane. The analysis of possible splice variants of arginase-I and arginase-II (http://www.ensembl.org/; http://www.genome.ucsc.edu/; http://www.ncbi.nlm.nih.gov/nucest/) predicts six (ENST00000368087, ENST00000469293, ENST00000356962, ENST00000275196, ENST00000484820, and ENST00000498260) variants of arginase-I in human. However,* ARG I* seems to have four transcripts variants (ENST00000261783, ENST00000557120, ENST00000556491, and ENST00000557319) in human. The splice variant analysis of* Mus musculus ARG II* (NM_009705) predicted four high scored splice sites at 261 bp, 388 bp, 407 bp, and 455 bp with scores of 8.571, 7.898, 7.542, and 9.505, respectively, by ASSP server. For* ARG I* (NM_007482), three high scored splice sites at 240 bp, 346 bp, and 378 bp with scores of 9.402, 7.223, and 7.612, respectively, were predicted by ASSP. Similarly, EST sequences of* ARG II* were observed in the range from 300 bp to 1024 bp. The virtual translation products of 1024 bp (DV039756) encode 197aa, 965 bp (DV074609), and 308aa. Observations provide us insight into the fact that the PCR products of* ARG I* (1 kb, 800 bp, and 300 bp) and arginase-II (1.2 kb, 700 bp, and 300 bp) could be the splice variants of arginase.

The physicochemical analysis and posttranslational modification of arginase-I and arginase-II were summarised in Table 1. Several posttranslational sites such as phosphorylation, glycosylation, N-O glycosylation, and acetylation sites were predicted in arginase-I and arginase-II. Analysis through MitoProt-II and iPSORT shows signal peptide “MFLRSSASRLLHGQIPCVLTRSVHSVAIVG” in arginase-II but not in arginase-I that is essential for the targeting of arginase-II to mitochondria. The analysis of virtual mutagenesis of the predicted targeting sequence of arginase-II revealed that amino acid residues from N-terminus “MFL.” do not affect targeting. However, arginine (R), serine (S), and stretch of seven residues “SRLLHGQ” out of “MFLRSSASRLLHGQIPCVLTRSVHSVAIVG” are very critical. The cleavage site between “MFLRSSASRLL.” and “HGQIPCVLTRSVHSVAIVG” was also identified. Such alterations in mitochondrial targeting sequences of arginase-II may alter transport and localization of arginase-II either on inner or on outer membrane of mitochondria (Figure 8).

### 3.4. Ultrastructural Changes Were Observed after KCl Treatment to Mitochondria

**Figure 7 fig7:**
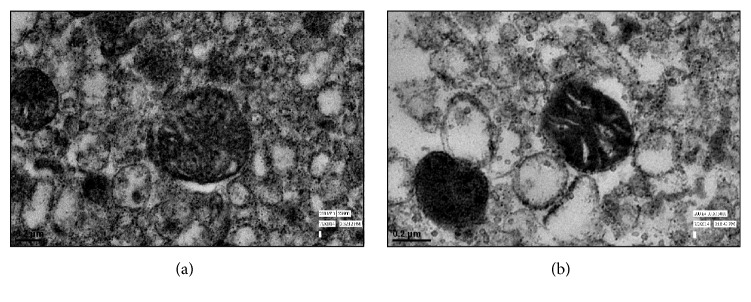
Transmission electron microscopic analysis of mitochondrial surface before and after the treatment of KCl showed mitochondrial swelling, altered morphology of cristae, and disruption in outer mitochondrial membrane after KCl treatment. (a) Ultrastructure of control mitochondria and (b) ultrastructure of KCl-treated mitochondria.

The analysis by TEM indicates KCl-dependent swelling of mitochondria and disruption of the outer mitochondrial membrane (Figure 7(a)). The deformities in the cristae of KCl-treated mitochondria (Figure 7(b)) were evident as compared to control.

## 4. Discussion and Conclusion

**Figure 8:**
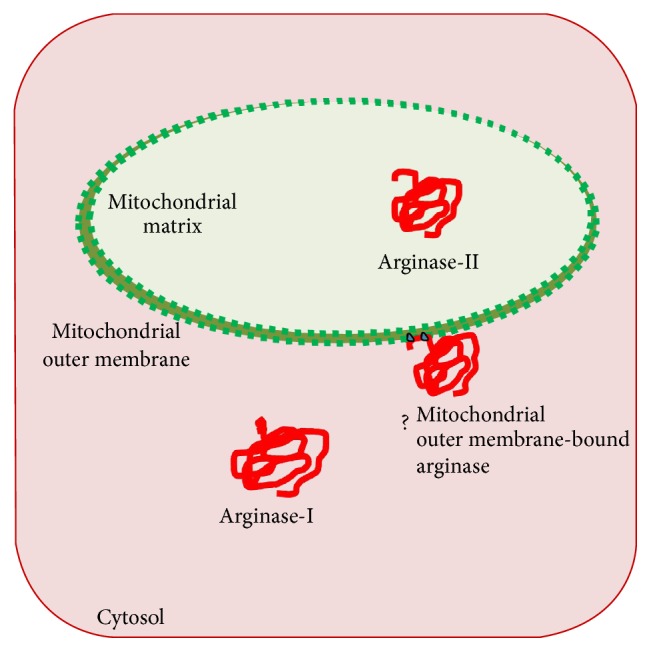
Illustration of presumed state of mitochondrial membrane-bound arginase. The question mark indicates the presence of mitochondrial membrane-bound arginase that may have been mistargeted due to posttranslational modification or alterations in the signal peptide.

The arginase-I facilitates removal of nitrogenous wastes and also serves as competitor substrate of the nitric oxide synthetase regulating nitric oxide production. However, mitochondrial arginase-II necessitates production of ornithine, proline, and glutamate required for synthesis of proteins and collagen. Since both isoforms get localised in different subcellular locations, they can efficiently utilize different pool of substrate. They help in efficient channelling of arginine and ornithine from mitochondria to cytosol and vice versa. Since transporters of arginine and ornithine have been found localised on the mitochondrial membrane along with K^+^ channels, the KCl may induce alterations in opening of mitochondrial permeability transition pores. Observations suggest KCl-dependent release of arginase and also effect of KCl on mitochondrial integrity. Results provided insight about proteins released from mitochondria because the mitochondria were isolated first without KCl in homogenizing medium; after that the mitochondrial pellet was washed with 0–200 mM KCl in homogenizing medium (Figure 5 [Fig fig6] [Fig fig7]). Arginase activity in KCl-washed fractions suggested that the mitochondrial membrane-bound arginase could be the mitochondrial arginase released under influence of KCl (Figure 8). This specific polypeptide could be a variant of arginase-II (Figure 3(b)) and is likely to be presumed mitochondrial membrane-bound fraction. Since the second wash (W2) removed all the disrupted mitochondrial content, the band detected in the membrane-bound fraction was released under influence of the KCl. Observations (Figures 3 [Fig fig4]–5) also confirmed that the increasing mitochondrial arginase activity in membrane-bound fraction leaked as the membrane-bound fraction from the mitochondrial matrix. Findings also indicate that the arginase-I does not remain associated with membrane of mitochondria. If it would have been so, the levels of arginase-I would have been higher, not arginase-II. Higher level of arginase activity under influence of KCl seems to be due to the leakage of mitochondrial arginase-II because activity of arginase-II got decreased and intensity of arginase-II reactive band increased. The decrease in the mitochondrial matrix arginase-II confirms the leakage of mitochondrial enzyme at higher concentration of KCl. It seems that mitochondrial membrane-bound arginase and arginase-II contain similar epitope but may differ in charge or get unique molecular signature due to posttranslational modifications. Earlier, different isoforms of arginase have also been reported [13, 14, 16, 17] but our results suggest that the mitochondrial membrane-bound arginase could be mitochondrial arginase, not the cytosolic arginase (Figure 3). A similar observation [32] also reveals that arginase associated with plasma membrane did not show cross reactivity with the liver cytosolic arginase.

The* in silico* analysis of arginase-I and arginase-II with MitoProt (http://ihg.gsf.de/ihg/mitoprot.html) [33] and iPSORT (http://ipsort.hgc.jp/predict.cgi) [34] also suggested that critical amino acid residues arginine (R), serine (S), and stretch of seven residues “SRLLHGQ” in the signal peptide of arginase-II seem critical for targeting. Since cleavage between “MFLRSSASRLL.” and “HGQIPCVLTRSVHSVAIVG” may lead to mistargeting of arginase-II to other organelles, it is likely that posttranslational modifications, particularly phosphorylation to serine (S), proline (P), and threonine (T) residues, may alter targeting to mitochondrial matrix and remain associated with the membrane (Table 1). Such phosphorylated arginase-II might have altered mobility (Figure 5(d)) and is detected as a variant of arginase-II associated with mitochondrial outer membrane (Figure 8). The* in silico* prediction of posttranslational modification also suggests the probability of phosphorylation at serine and threonine residues (http://www.expasy.org/proteomics). Not only phosphorylation but also glycosylation sites were predicted in arginase-II protein sequence that may alter the mobility of arginase and association with mitochondrial membrane. The important spliced variants and/or ESTs of* ARG I* and* ARG II* ranging from 132 bp (AW113714) to 1,166 bp (BI649622) also support the idea of possible splice variant. The virtual translation of matching UniGene sequences DV039756 and DV074609 provides insight that the PCR products (Figure 6) of* ARG I* (1 kb, 800 bp, and 300 bp) and* ARG II* (1.2 kb, 700 bp, and 300 bp) could be the splice variants of arginase.

Thus, mitochondrial-membrane bound arginase seems similar to arginase-II, a novel variant, or could be arginase-II having posttranslational modifications which is localized to mitochondria for a specialized function.

## Figures and Tables

**Table 1:** Comparative properties of *Mus musculus* arginase-I and arginase-II.

Parameters	Arginase-I (AAA98611.1)	Arginase-II (AAC22548.1)
Molecular weight	34807.8	38878.3
pI	6.52	6.10
Total number of negatively charged residues (Asp + Glu)	39	40
Total number of positively charged residues (Arg + Lys)	37	33
Aliphatic index	90.77	97.46
Grand average of hydropathicity (GRAVY) (http://www.expasy.org/proteomics)	−0.187	−0.109
Mitochondrial import (https://ihg.gsf.de/ihg/mitoprot.html)	No (0.060)	Yes (0.9771)
N-phosphorylation sites (http://www.cbs.dtu.dk/services/NetPhos/)	20 sites (9 serine residues, 6 threonine residues, and 5 tyrosine residues)	18 sites (one site S^6^ (0.974) in mitochondrial targeting sequence)
N-acetylation sites (http://www.cbs.dtu.dk/services/NetAcet/)	2 sites at 2nd S (score 0.501) and 3rd S (score 0.492) from N-terminus	No acetylation sites were found from N-terminus
O-GlcNAcetylation sites (http://www.cbs.dtu.dk/services/YinOYang/)	9 sites	7 sites (no sites were found in mitochondrial targeting sequence)
N-glycosylation sites (http://www.cbs.dtu.dk/services/NetNGlyc/)	5 sites	5 sites (no sites in mitochondrial targeting sequence)
